# Supplementary figures and images for: R-locus for roaned coat is associated with a tandem duplication in an intronic region of USH2A in dogs and also contributes to Dalmatian spotting
Source: PLoS One. 2021 Mar 23;16(3):e0248233. doi: 10.1371/journal.pone.0248233 (PMC7987146; doi:10.1371/journal.pone.0248233)

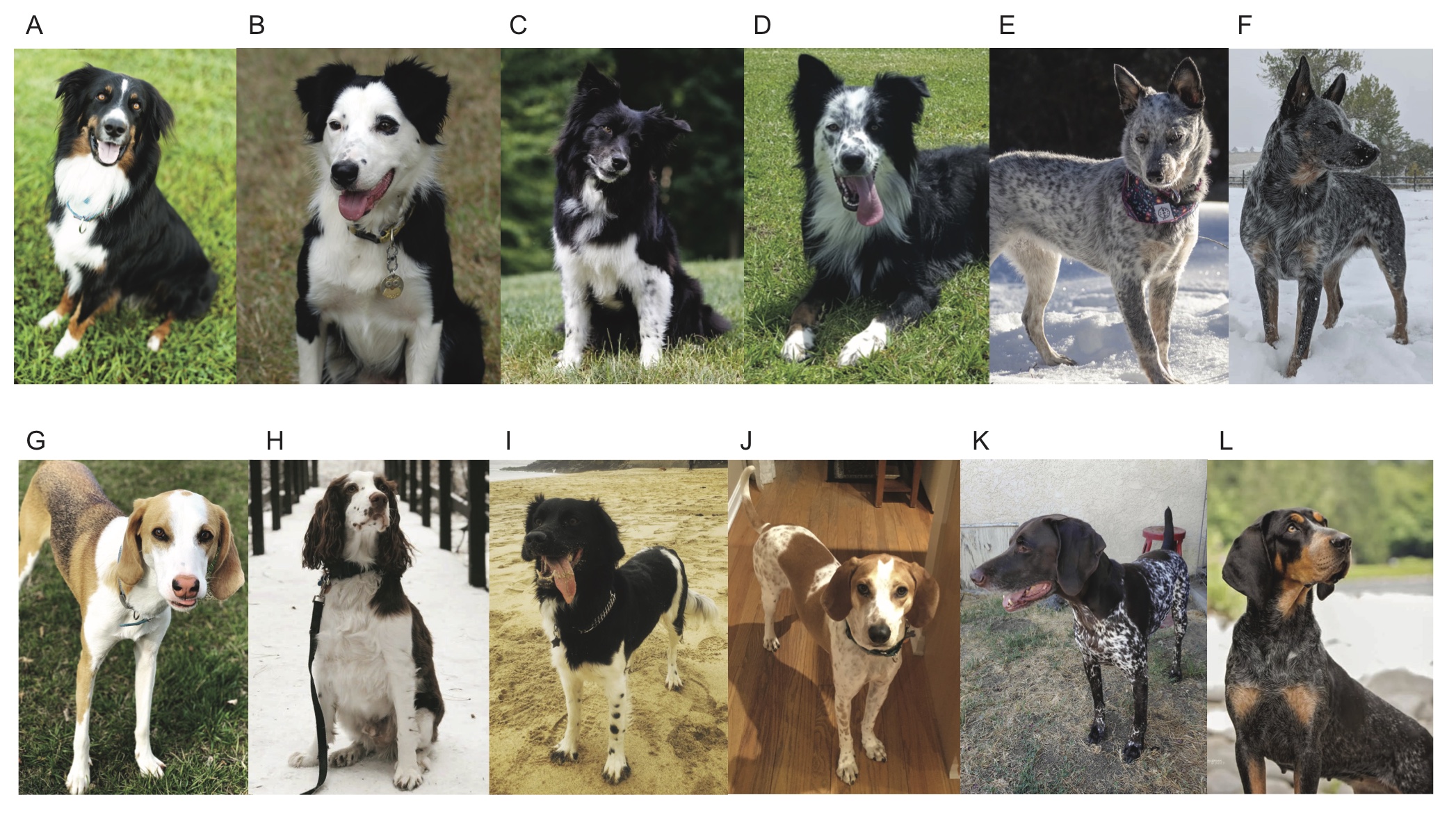

Supplement: S1 Fig — A) and G) No ticking. B), C), H), and I) Lightly ticked. D) and J) Heavily ticked. E), F), K) and L) Heavily ticked but also roaned. Dogs with ticking and roaning were excluded from the study. Photo credit (from A to L): Gusto and Chris Cameron Sarafin, Hootenanny and Deanna Haase, Lira and Judi Schachte, Hugo and Kirby Brannon, Jetta and W.Fluckey/C.Moore, Darwin and Colin Scott, Paco Muñoz-Scaggs and Diana Muñoz-Scaggs, Martin and Brittney Mitchell, Nova Fox Bijou Duchesse de Hedwige and K. Smith, Joey and Marissa Crean, Hans and Kari Cueva, CH Cedar Creek’s I’m So Fancy and Brittany Fischer. (JPG) [file pone.0248233.s001.jpg]

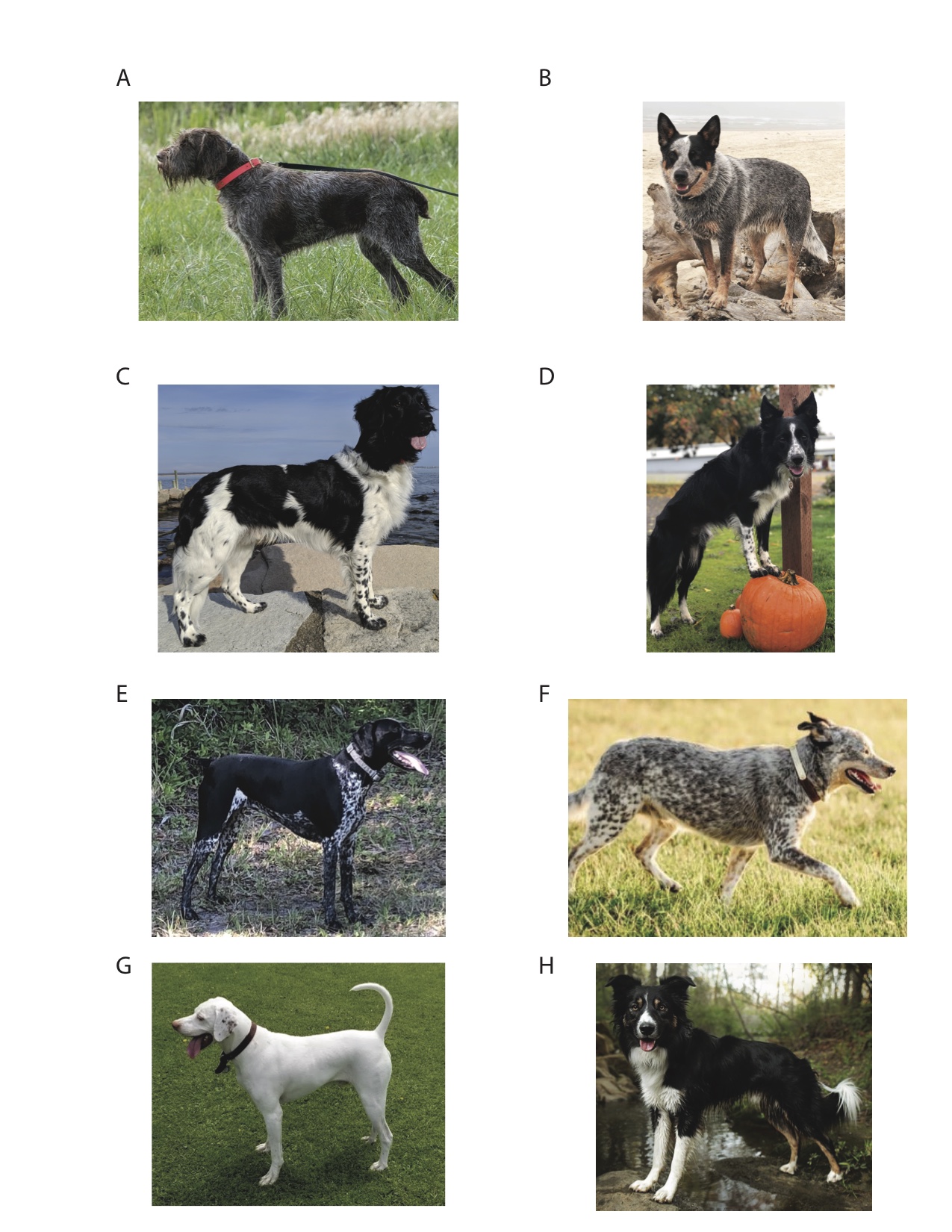

Supplement: S2 Fig — A) Spinone Italiano (roaned). B) Australian Cattle Dog (roaned). C) Stabyhoun (ticked). D) Border Collie (ticked). E) German Shorthaired Pointer (both roaned and ticked). F) Australian Cattle Dog (both roaned and ticked). G) Pointer (without roaning and ticking). H) Border Collie (without roaning and ticking). A, C, E, and G are non- herding breeds, while B, D, F, and H are herding breeds. Photo credit (from A to H): A. Barber, Alexis Q., E. Nado, Heather O’Neill, Chris and Barbara T., G. C., Erica Murray, and Adriana N. (JPG) [file pone.0248233.s002.jpg]

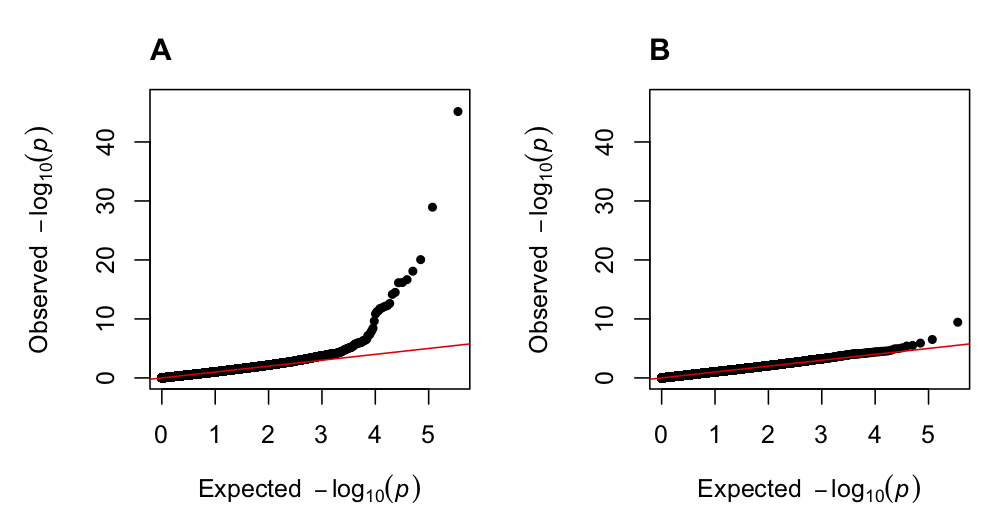

Supplement: S3 Fig — Q-Q plots of the association with A) roaning and B) ticking. (PNG) [file pone.0248233.s003.png]

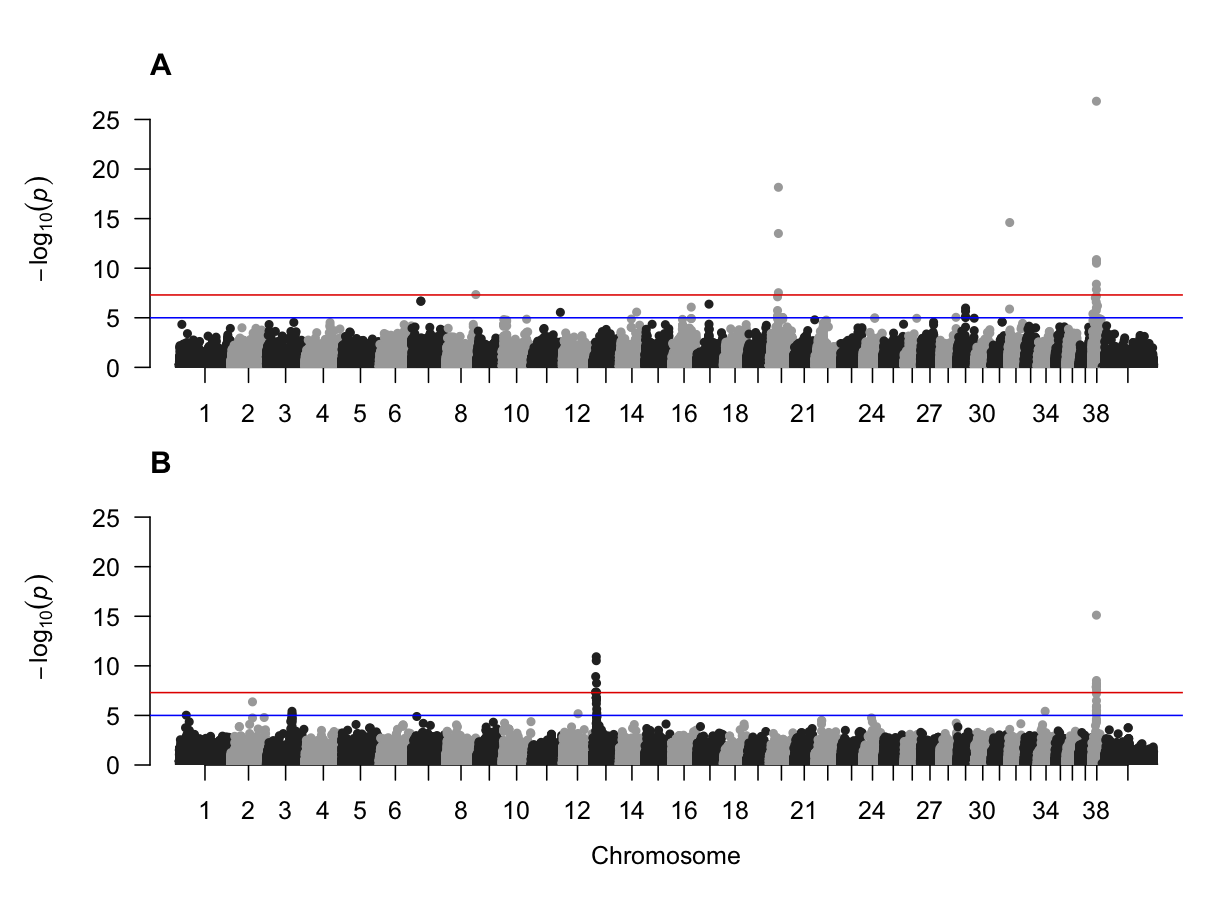

Supplement: S4 Fig — A) Herding breeds. B) Non-herding breeds. Red and blue horizontal lines are significant (P < 5 x 10^−8^) and suggestive (P < 1 x 10^−5^) associations, respectively. (PNG) [file pone.0248233.s004.png]

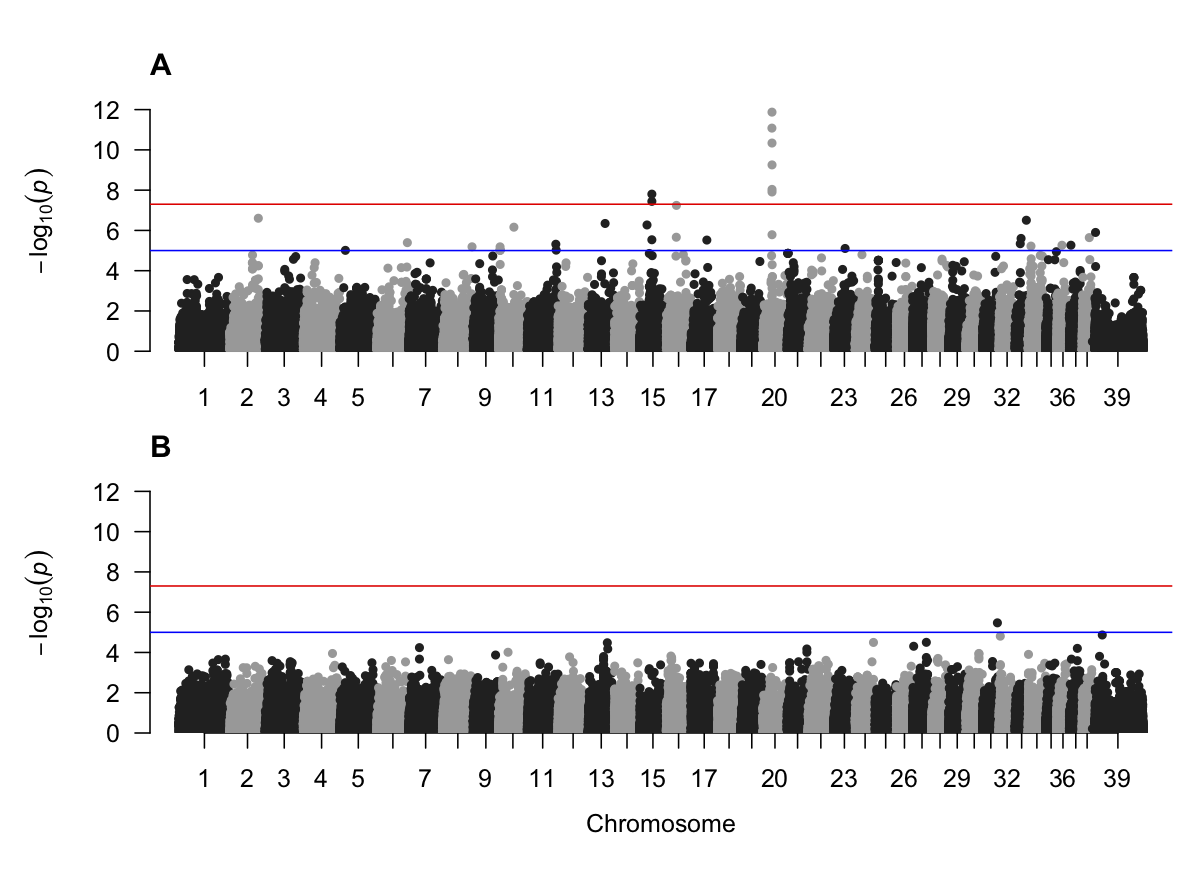

Supplement: S5 Fig — A) Herding breeds. B) Non-herding breeds. Red and blue horizontal lines are significant (P < 5 x 10^−8^) and suggestive (P < 1 x 10^−5^) associations, respectively. (PNG) [file pone.0248233.s005.png]

Read depth

Australian Cattle Dog  
(SRR7107580)

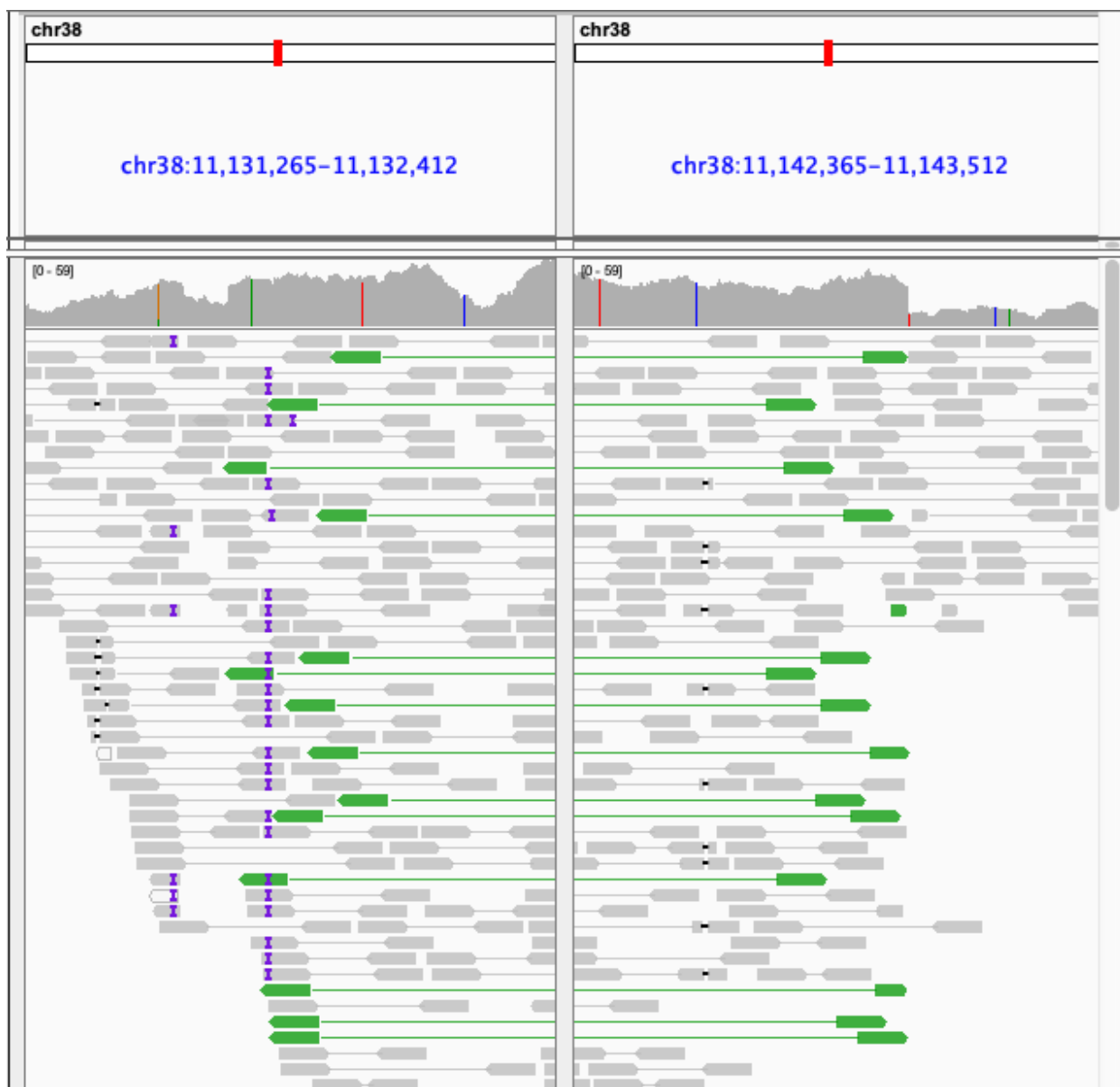

Read depth

Boarder Collie  
(SRR7107950)

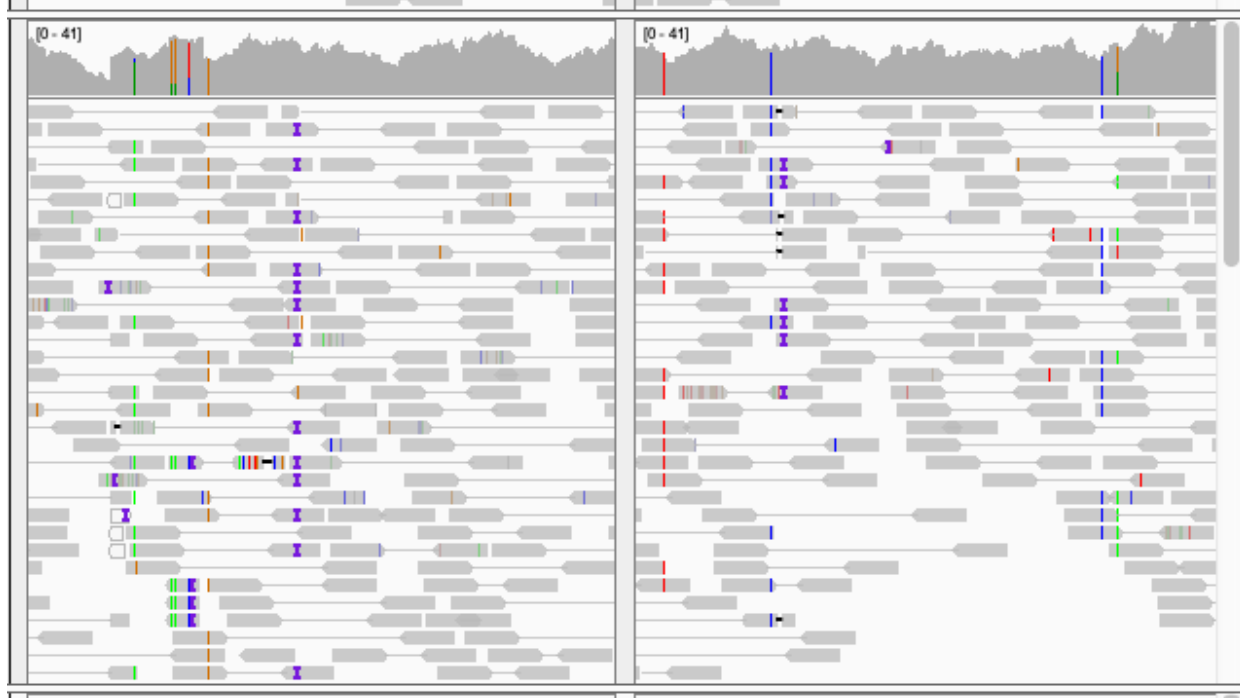

Supplement: S6 Fig — Discordant read pairs at the duplication breakpoint on CFA38 identified in Australian Cattle Dog (top panel), and Border Collie (bottom panel). Outward-facing read pairs (green) indicate that this is a tandem duplication found in Australian Cattle Dog (usually roaned) but not in Border Collie. (PDF) [file pone.0248233.s006.pdf]

A

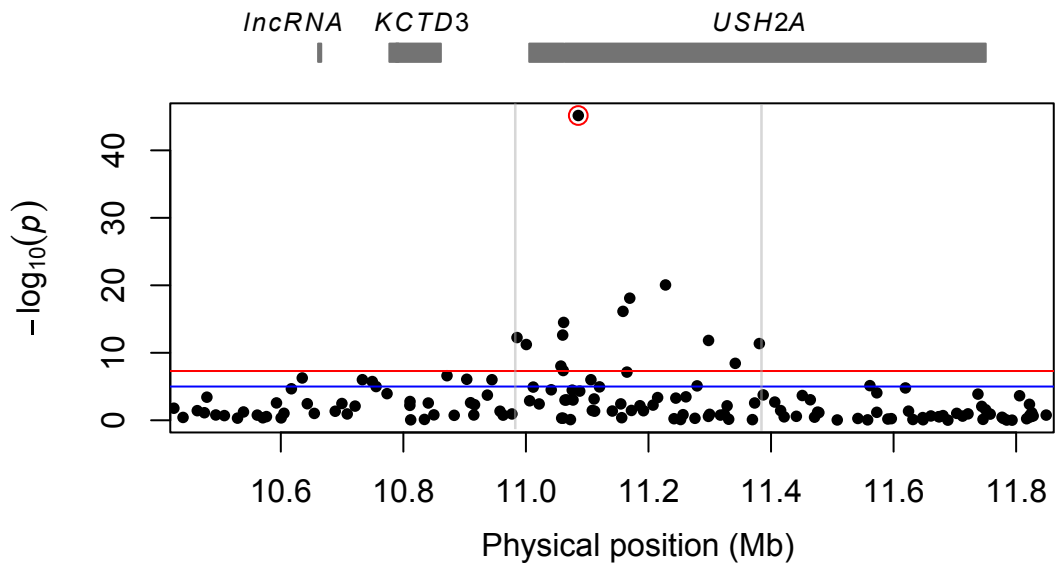

B

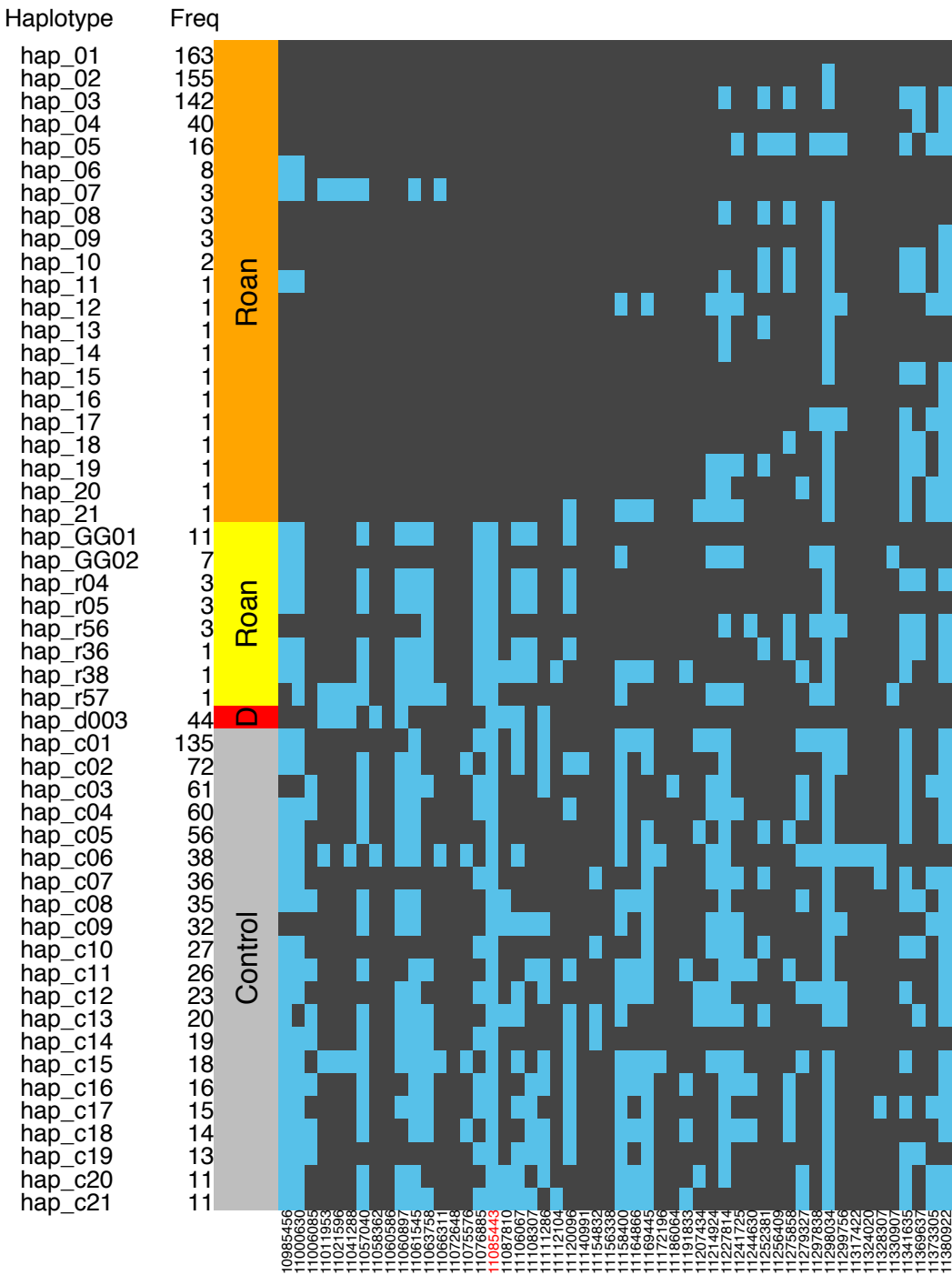

Supplement: S7 Fig — A) Manhattan plot of association with roaning on chromosome 38. Red and blue horizontal lines are significant (P < 5 x 10^−8^) and suggestive (P < 1 x 10^−5^) associations, respectively. Genes are indicated in grey boxes. Grey vertical lines indicate the region indicated in the panel B. B) Haplotypes defined by 52 single nucleotide variant (SNV) markers in the roan-associated region (CFA38:10,985,456–11,380,922) and their frequencies. Rows correspond to haplotypes, and columns correspond to markers. Haplotypes in orange (hap_01—hap_21) have the roan-associated “A” allele at the most significant marker (CFA38:11,085,443). Eight haplotypes in yellow are identified in roaned dogs without the roan-associated “A” allele at the most significant marker. A haplotype in red was found in Dalmatians. Haplotypes in grey were identified in control groups without roaning. Only the haplotypes that are mentioned in the main text or with frequencies larger than 10 are shown (see S5 Table for the full list of haplotypes). (PDF) [file pone.0248233.s007.pdf]

A

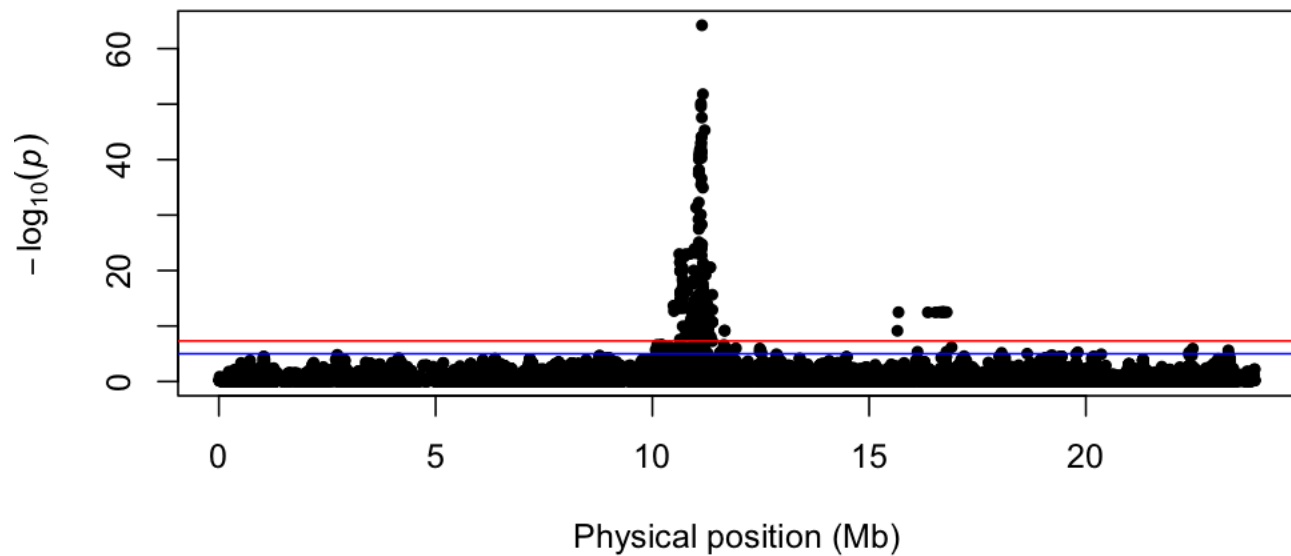

B

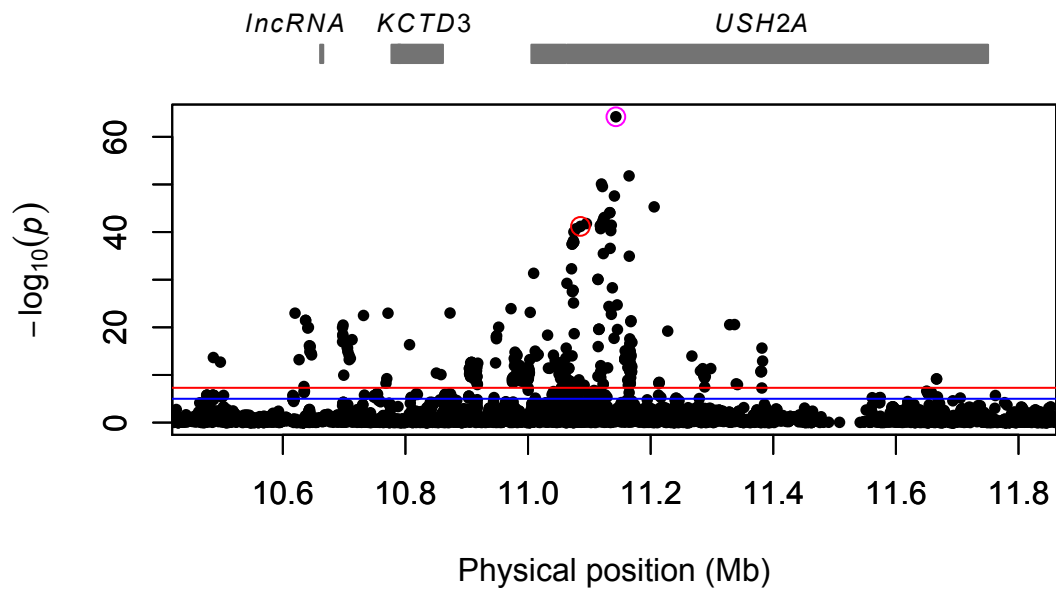

Supplement: S8 Fig — A) Entire chromosome. B) A region close to the most significant marker (magenta circle). The most significant GWAS marker is in red circle. The most significant CWAS marker is in magenta circle. Red and blue horizontal lines are significant (P < 5 x 10^−8^) and suggestive (P < 1 x 10^−5^) associations, respectively. (PDF) [file pone.0248233.s008.pdf]

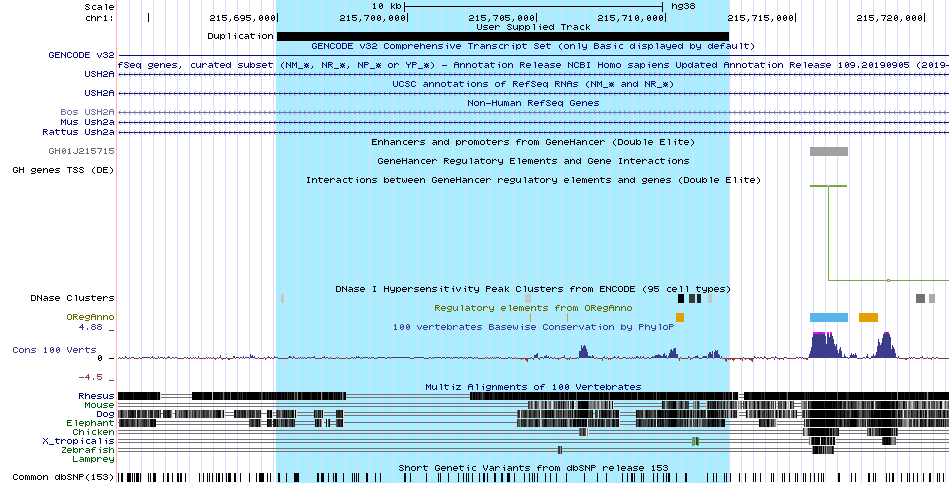

Supplement: S9 Fig — The highlighted area in blue is the orthologous region to the tandem duplication identified in dogs with roaning, which is located within the intron 61 of USH2A. GeneHancer Regulatory Elements are located at chr1:215,715,579–215,717,032 (green line), which corresponds to CFA38:11,146,170–11,147,605 in the dog genome (CanFam3.1). DNAse I hypersensitive sites: grey and black boxes. Open Regulatory Annotation (ORegAnno): orange and blue boxes. Cons 100 Verts (100 vertebrates basewise conservation by PhyloP score): blue histogram. (PNG) [file pone.0248233.s009.png]

No haplotype

Heterozygote

Homozygote

Roaned  
Not Roaned

-0.6

-0.4

-0.2

0.0

0.2

0.4

0.6

$\Delta\text{LRR}$

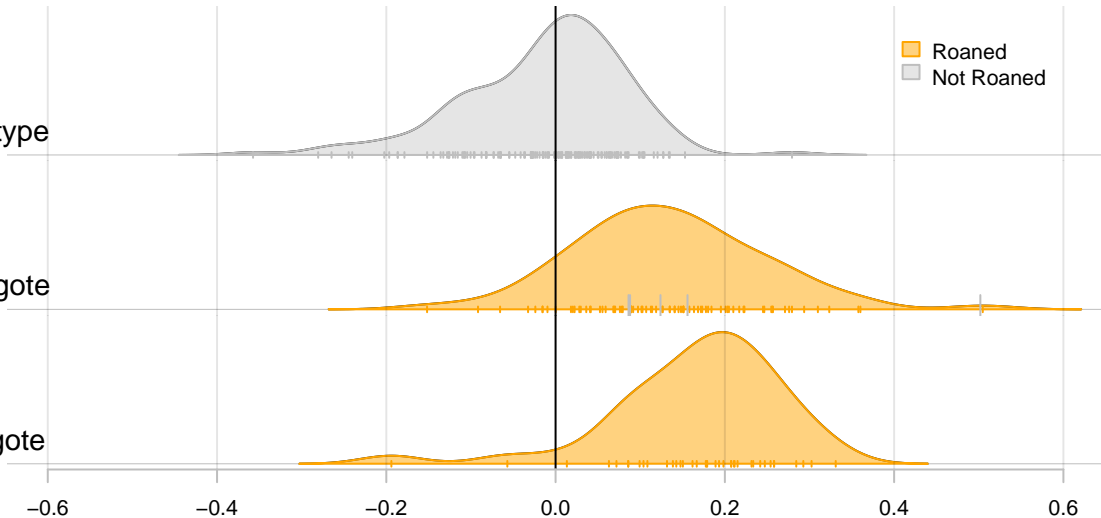

Supplement: S10 Fig — Vertical ticks indicate individual ΔLRR of dogs with roaning (orange) and without roaning (grey). Density plots with the number of individuals less than 10 are not shown, but individual ΔLRR is indicated with longer vertical ticks. (PDF) [file pone.0248233.s010.pdf]

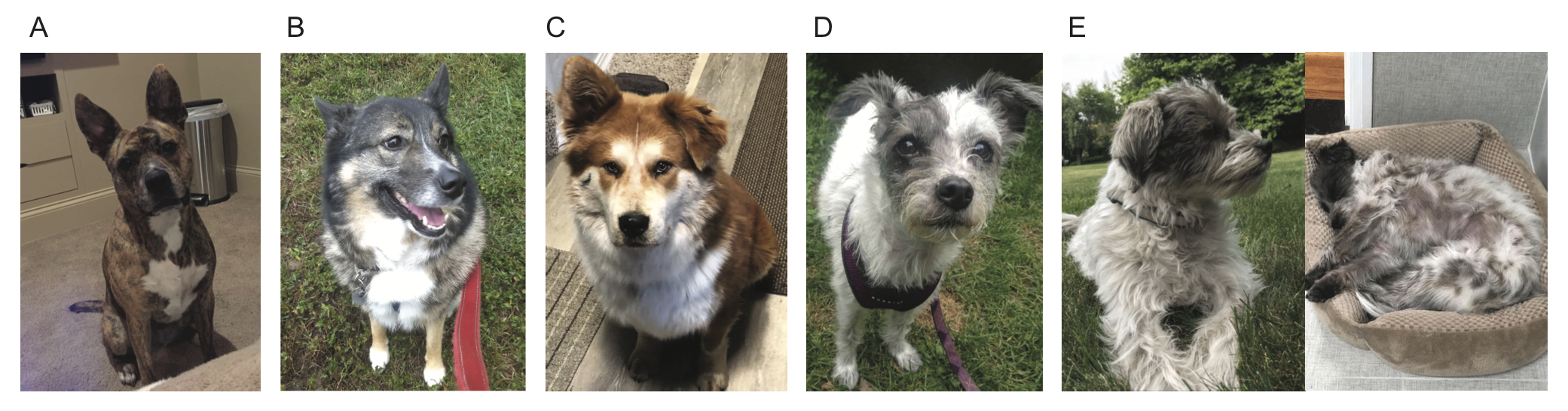

Supplement: S11 Fig — These five dogs carry a duplication-associated haplotype but show no or little roaned coat. In the panel E, roan and/or tick is invisible when fur is long (left) but is visible when the coat is shaved (right). Photo credit (from A to E): William DeLozier, Dorothy Olszewski, Thomas Borr, Rebeccah Kivitz, and Maria Casey. (PNG) [file pone.0248233.s011.png]

Normalized read depth

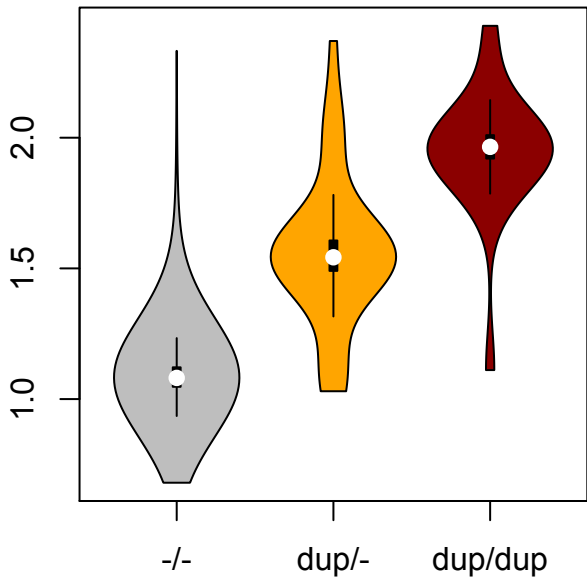

Supplement: S12 Fig — Mean variant read depth (DP) within the duplication was divided by the mean variant DP of the flanking 100-kb region for normalization. (PDF) [file pone.0248233.s012.pdf]

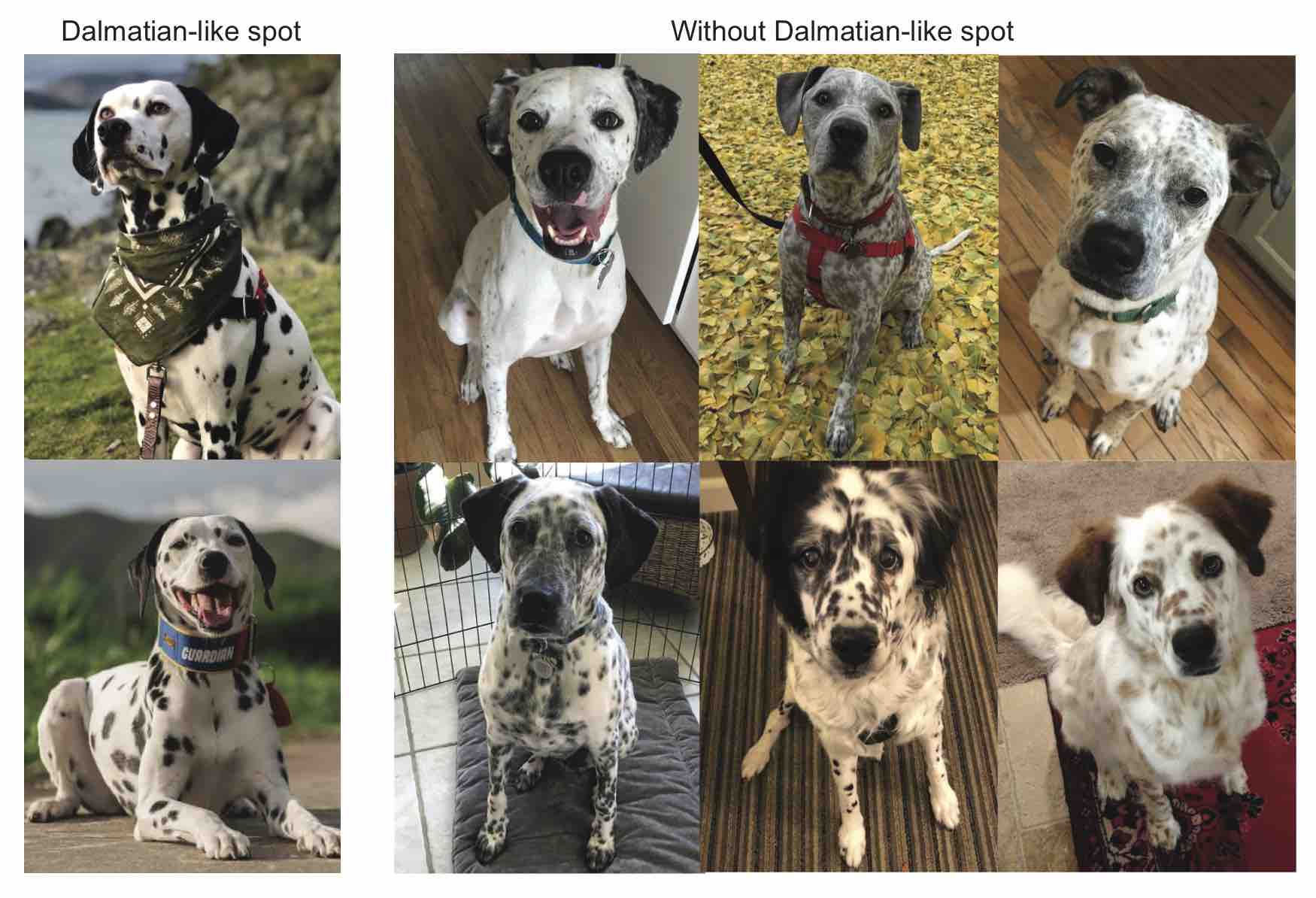

Supplement: S13 Fig — Photo credit (clockwise from top-left): Gina Vrdoljak, Lindsay Jakobovits, Darragh Nolan, Catharine Giannasi, Ann Holland, Susie Johnston, Laurence Montgomery, and Raquel Lorenzo. (JPG) [file pone.0248233.s013.jpg]
