# Supplementary material for: R-locus for roaned coat is associated with a tandem duplication in an intronic region of USH2A in dogs and also contributes to Dalmatian spotting
Source: PLoS One. 2021 Mar 23;16(3):e0248233. doi: 10.1371/journal.pone.0248233 (PMC7987146; doi:10.1371/journal.pone.0248233)
Supplement: S3 Table — (A) Primer sequences used for PCR assays described in Fig 4. The expected sizes of the PCR products are 539 bp (Tick38-F1/Tick38-R1), 476 bp (Tick38-F2-2/Tick38-R2-2), and 397 bp (Tick38-F2-2/ Tick38-R1). The former two pairs amplify franking region of the CFA38 duplication (positive controls), whereas the last pair amplifies the midpoint of the duplication. (B) Midpoint span product sequence. Nucleotides in bold and italic are likely the end of the first copy and the beginning of the second copy, respectively. (DOCX) [file pone.0248233.s016.docx]

**S3 Table. (A) Primer sequences used for PCR assays described in Fig 4. The expected sizes of the PCR products are 539 bp (Tick38-F1/Tick38-R1), 476 bp (Tick38-F2-2/Tick38-R2-2), and 397 bp (Tick38-F2-2/ Tick38-R1).** The former two pairs amplify franking region of the CFA38 duplication, whereas the last pair amplifies the midpoint of the duplication. (B) Midpoint span product sequence. Nucleotides in bold and italic are likely the end of the first copy and the beginning of the second copy, respectively.

**A.**

| Primer Name | Sequence | Priming position |
| --- | --- | --- |
| Tick38-F1 | ATTCAGCATCATGGAGCCCC | CFA38:11,131,450-11,131,469 |
| Tick38-R1 | TCACCCATCCTTTCACCCAC | CFA38:11,131,969-11,131,988 |
| Tick38-F2-2 | CAGCAATGGTGGGGAAGCTA | CFA38:11,142,992-11,143,011 |
| Tick38-R2-2 | GAGTCCAGGTGTCACAGTGG | CFA38:11,143,448-11,143,467 |

**B.**

**CAGCAATGGTGGGGAAGCTAATTTTAGGTCTACATTAGGTTTCTTTGTAAATCAGAAATTGCACCAGGTGACATAAGGAAAGATGGCAGCGTGGGTCCTGGTATAGAGCCTGCCTATGAGACAATTGCAGAAGAGAGATCCACTCTACCTTCCCTCCTGCTCCTTAAGGGACTCTTGGGGCATCTCAAGGGCAGGTGTTGGAGGGGTGCCCTAACGGTCAGTGCTGTTTCTGAATGCTGATGA***AAATAAGTTAATCAGAGAAAGACAAATACCATATGATTTCATTCATGTGGAATTTAAGAAACAAAACAAATGAACAAAGGAAAAAAAAAAGACTCTTACCTATACAGAACAAACAGATGATTACCAGAAGAAAGGTGGGTGAAAGGATGGGTGA*
