# Supplementary material for: R-locus for roaned coat is associated with a tandem duplication in an intronic region of USH2A in dogs and also contributes to Dalmatian spotting
Source: PLoS One. 2021 Mar 23;16(3):e0248233. doi: 10.1371/journal.pone.0248233 (PMC7987146; doi:10.1371/journal.pone.0248233)
Supplement: S4 Table — (A) The top CWAS marker associated with roaning (CFA38:11,143,243). (B) A candidate F-locus marker associated with Dalmatian’s spot (CFA3:72,316,930). (DOCX) [file pone.0248233.s017.docx]

**S4 Table. Primer sequences used for PCR to genotype SNPs associated with coat color phenotype.** (A) The top CWAS marker associated with roaning (CFA38:11,143,243). (B) A candidate F-locus marker associated with Dalmatian’s spot (CFA3:72,316,930).

**A.**

| Primer Name | Sequence | Priming position |
| --- | --- | --- |
| CWAS38-F1 | GACTCTTGGGGCATCTCAAGG | CFA38:11,143,161-11,143,181 |
| CWAS38-R1 | CATGACCACAGACAGTGCTG | CFA38:11,143,306-11,143,326 |

**B.**

| Primer Name | Sequence | Priming position |
| --- | --- | --- |
| Dal3-F1 | CCTCCCCAAGAATATAACTGTTACAC | CFA3:72,316,831-72,316,856 |
| Dal3-R1 | TCATATTGTCATGGAGAGAAATGCACG | CFA3:72,317,105-72,317,131 |
