# Supplementary material for: R-locus for roaned coat is associated with a tandem duplication in an intronic region of USH2A in dogs and also contributes to Dalmatian spotting
Source: PLoS One. 2021 Mar 23;16(3):e0248233. doi: 10.1371/journal.pone.0248233 (PMC7987146; doi:10.1371/journal.pone.0248233)
Supplement: S6 Table — A) CFA38 Duplication. B) The top associated CWAS marker based on the imputed genotypes (CFA38:11,143,243). C) The top associated GWAS marker (CFA38:11,085,443). D) CFA38 Duplication and the top associated CWAS in roaned dogs. E) A missense mutation at CFA38:11,111,286 based on the imputed genotypes. F) A missense mutation at CFA38:11,169,445 based on the imputed genotypes. (DOCX) [file pone.0248233.s019.docx]

**S6 Table. Genotype frequencies of the markers associated with roaning in the discovery panel.** A) CFA38 Duplication. B) The top associated CWAS marker based on the imputed genotypes (CFA38:11,143,243). C) The top associated GWAS marker (CFA38:11,085,443). D) CFA38 Duplication and the top associated CWAS in roaned dogs. E) A missense mutation at CFA38:11,111,286 based on the imputed genotypes. F) A missense mutation at CFA38:11,169,445 based on the imputed genotypes.

A) CFA38 Duplication

|  | +/+ | +/- | -/- |
| --- | --- | --- | --- |
| Roaned | 246 | 112 | 0 |
| Non-roaned | 0 | 0 | 579 |

+/+: homozygotes for the duplication

+/-: heterozygotes for the duplication

-/-: no duplication

B) Top associated CWAS marker

|  | T/T | T/C | C/C | 0/0 |
| --- | --- | --- | --- | --- |
| Roaned | 251 | 94 | 0 | 13 |
| Non-roaned | 0 | 0 | 579 | 0 |

0/0: Genotypes not imputed with probability >90 %

C) Top associated GWAS marker

|  | A/A | A/G | G/G |
| --- | --- | --- | --- |
| Roaned | 204 | 138 | 18 |
| Non-roaned | 0 | 0 | 579 |

D) CFA38 duplication and the top associated CWAS marker in roaned dogs. All non-roaned dogs did not have the duplication (-/-) and were T/T at the CWAS marker (N = 579)

|  | T/T | T/C | C/C | 0/0 |
| --- | --- | --- | --- | --- |
| +/+ | 246 | 0 | 0 | 0 |
| +/- | 5 | 94 | 0 | 13 |
| -/- | 0 | 0 | 0 | 0 |

+/+: homozygotes for the duplication

+/-: heterozygotes for the duplication

-/-: no duplication

0/0: Genotypes not imputed with probability >90 %

E) Missense mutation at CFA38:11,111,286

|  | G/G | G/A | A/A | 0/0 |
| --- | --- | --- | --- | --- |
| Roaned | 0 | 36 | 322 | 0 |
| Non-roaned | 136 | 278 | 165 | 0 |

0/0: Genotypes not imputed with probability >90 %

A: Missense variant, introducing GAA/AAA (Glu/Lys) substitution

F) Missense mutation at CFA38:11,169,445

|  | C/C | C/T | T/T | 0/0 |
| --- | --- | --- | --- | --- |
| Roaned | 0 | 74 | 284 | 13 |
| Non-roaned | 333 | 198 | 48 | 0 |

0/0: Genotypes not imputed with probability >90 %

T: Missense variant, introducing CCT/TCT (Pro/Ser) substitution
