# Supplementary material for: R-locus for roaned coat is associated with a tandem duplication in an intronic region of USH2A in dogs and also contributes to Dalmatian spotting
Source: PLoS One. 2021 Mar 23;16(3):e0248233. doi: 10.1371/journal.pone.0248233 (PMC7987146; doi:10.1371/journal.pone.0248233)
Supplement: S7 Table — (DOCX) [file pone.0248233.s020.docx]

**S7 Table. Genotype frequencies of the CFA38 duplication associated with roaning and the imputed genotype at CFA38:11,143,243 in the validation panel.**

A) CFA38 duplication

|  | +/+ | +/- | -/- |
| --- | --- | --- | --- |
| Roaned | 40 | 80 | 0 |
| Non-roaned | 0 | 5 | 149 |

+/+: homozygotes for the duplication

+/-: heterozygotes for the duplication

-/-: no duplication

B) Genotype frequency of CFA38:11,143,243

|  | T/T | T/C | C/C | 0/0 |
| --- | --- | --- | --- | --- |
| Roaned | 40 | 79 | 0 | 1 |
| Non-roaned | 0 | 5 | 149 | 0 |

0/0: Genotypes not imputed with probability >90 %
