# Supplementary material for: R-locus for roaned coat is associated with a tandem duplication in an intronic region of USH2A in dogs and also contributes to Dalmatian spotting
Source: PLoS One. 2021 Mar 23;16(3):e0248233. doi: 10.1371/journal.pone.0248233 (PMC7987146; doi:10.1371/journal.pone.0248233)
Supplement: S9 Table — A) Genotype frequency at CFA3:69,456,869. B) Genotype frequency at CFA3:72,316,930 based on the imputed genotypes. Roaned dogs were purepred dogs used for GWAS (the discovery panel dogs). (DOCX) [file pone.0248233.s022.docx]

**S9 Table. Genotype frequencies at the causal variant of hyperuricosuria at CFA3:69,456,869 and CFA3:72,316,930 in purebred Dalmatian, roaned dogs, and mixed breeds with or without Dalmatian-like spots.** A) Genotype frequency at CFA3:69,456,869. B) Genotype frequency at CFA3:72,316,930 based on the imputed genotypes. Roaned dogs were purepred dogs used for GWAS (the discovery panel dogs).

A) CFA3:69,456,869

|  | C/C | C/A | A/A | 0/0 |
| --- | --- | --- | --- | --- |
| Dalmatian | 4 | 34 | 224 | 0 |
| Roaned | 355 | 3 | 0 | 0 |
| Mix with Dalmatian-like spots | 0 | 1 | 6 | 0 |
| Mix without Dalmatian-like spots | 10 | 24 | 2 | 0 |

0/0: Genotypes not imputed with probability >90 %

A: Risk allele for hyperuricosuria (HUU)

B) CFA3:72,316,930

|  | G/G | G/A | A/A | 0/0 |
| --- | --- | --- | --- | --- |
| Dalmatian | 0 | 6 | 256 | 0 |
| Roaned | 281 | 48 | 7 | 22 |
| Mix with Dalmatian-like spots | 0 | 0 | 7 | 0 |
| Mix without Dalmatian-like spots | 11 | 21 | 4 | 0 |

0/0: Genotypes not imputed with probability >90 %
