# Supplementary material for: R-locus for roaned coat is associated with a tandem duplication in an intronic region of USH2A in dogs and also contributes to Dalmatian spotting
Source: PLoS One. 2021 Mar 23;16(3):e0248233. doi: 10.1371/journal.pone.0248233 (PMC7987146; doi:10.1371/journal.pone.0248233)
Supplement: S1 Text — (PDF) [file pone.0248233.s033.pdf]

Genotype-phenotype association analysis by using imputed genotypes

To search for candidate causal variants or variants tightly linked with causal variants, genotypes of known variants on CFA38 reported in Plassais et al. [1] were imputed for 1,473 dogs (937 and 274 dogs in the discovery and validation datasets, as well as 262 dalmatians) by using IMPUTE2 version 2.3.2 [2] with 722 dogs and other canid species [1] as a reference panel. Imputed genotypes with the genotype probabilities less than 10 % were treated as missing (196,426 SNPs and 31,621 indels with mean missing genotypes = 15.6 % per variant). Similarly, genotypes of 604,843 SNPs on CFA3 were imputed by IMPUTE2 for dalmatians, roaned dogs, and mixed breed dogs (N = 262, 358, and 43, respectively) to identify markers associated with dalmatian spots. The accuracy of the imputed genotypes was evaluated by Sanger sequencing PCR fragments, which were generated by using primer pairs spanning the position of the imputed SNP (Table in S4 Table). The phenotype-genotype associations for the imputed marker on CFA38 were evaluated using GEMMA [3] with the same parameter setting described in the previous section.

Chromosome-wide association study (CWAS) by using the imputed genotypes of 228,047 bi-allelic sites on CFA38 (196,426 SNVs and 31,621 indels) found the most significant association at the position 11,143,243 (Figure in S8 Fig), which was 6-bp away from the duplication (CFA38:11,131,835-11,143,237). After excluding 13 dogs whose genotypes at this SNV were not imputed with probability of >90 %, 251 and 94 roaned dogs had imputed TT homozygous and TC heterozygous genotypes, respectively, whereas there was no dog with CC homozygous

genotype (Table in S6 Table). All control dogs were CC homozygotes at this site. In addition, all dalmatians had at least one copy of the T allele at this site (226 and 2 TT and TC genotypes, respectively; 34 dogs with genotype probability <90 %). Accuracy of the imputed genotypes was confirmed by Sanger sequencing the PCR-amplified fragment (N = 41) (Table in S4 Table).

To identify genomic regions associated with the Dalmatian's spot, we imputed genotypes of 604,843 SNVs on CFA3 reported in Plassais et al. [1] for 358 roaned dogs in the discovery dataset and 262 Dalmatians. Of these, genotypes of 581,891 SNVs were imputed with >90 % probability for >90 % of Dalmatians and roaned dogs. In this dataset, 34 and 224 Dalmatians had one or two copies of hyperuricosuria risk allele at CFA:69,456,869, while four Dalmatians did not have the risk allele (Table in S10 Table). Regardless of the genotype at CFA:69,456,869, all Dalmatians had typical Dalmatian spots. Comparison of genotype frequencies between Dalmatians and roaned dogs showed that there was no SNV where Dalmatians were fixed with one variant while roaned dogs were either heterozygous or homozygous of the alternate variant. A SNV at CFA3:72,316,930, which was 2.9 Mb away from the causal variant of hyperuricosuria, showed the largest genotype frequency difference between Dalmatians and roaned dogs, where 98 % of dalmatians and 2 % of roaned dogs were AA homozygotes (chi square test, $p = 1 \times 10^{-120}$) (Table in S10 Table). Six Dalmatians were GA heterozygotes, and none of them were homozygous for the reference G allele. This marker was in an intronic region of the Ras Homolog Family Member H (*RHOH*) gene.

References

1. Plassais J, Kim J, Davis BW, Karyadi DM, Hogan AN, Harris AC, et al. Whole genome sequencing of canids reveals genomic regions under selection and variants influencing morphology. Nat Commun. 2019;10: 1489. doi:10.1038/s41467-019-09373-w
2. Howie BN, Donnelly P, Marchini J. A Flexible and Accurate Genotype Imputation Method for the Next Generation of Genome-Wide Association Studies. PLOS Genet. 2009;5: e1000529. doi:10.1371/journal.pgen.1000529
3. Zhou X, Stephens M. Genome-wide efficient mixed-model analysis for association studies. Nat Genet. 2012;44: 821–824. doi:10.1038/ng.2310
